# E2-EPF UCP Possesses E3 Ubiquitin Ligase Activity via Its Cysteine 118 Residue

**DOI:** 10.1371/journal.pone.0163710

**Published:** 2016-09-29

**Authors:** Jung Hwa Lim, Hee Won Shin, Kyung-Sook Chung, Nam-Soon Kim, Ju Hee Kim, Hong-Ryul Jung, Dong-Soo Im, Cho-Rok Jung

**Affiliations:** 1 Gene Therapy Research Unit, Korea Research Institute of Bioscience and Biotechnology, Daejeon, Republic of Korea; 2 University of Science and Technology, Daejeon, Republic of Korea; 3 Genome Research Center, Korea Research Institute of Bioscience and Biotechnology, Daejeon, Republic of Korea; National University of Singapore, SINGAPORE

## Abstract

Here, we show that E2-EPF ubiquitin carrier protein (UCP) elongated E3-independent polyubiquitin chains on the lysine residues of von Hippel-Lindau protein (pVHL) and its own lysine residues both *in vitro* and *in vivo*. The initiation of the ubiquitin reaction depended on not only Lys11 linkage but also the Lys6, Lys48 and Lys63 residues of ubiquitin, which were involved in polyubiquitin chain formation on UCP itself. UCP self-association occurred through the UBC domain, which also contributed to the interaction with pVHL. The polyubiquitin chains appeared on the N-terminus of UCP *in vivo*, which indicated that the N-terminus of UCP contains target lysines for polyubiquitination. The Lys76 residue of UCP was the most critical site for auto-ubiquitination, whereas the polyubiquitin chain formation on pVHL occurred on all three of its lysines (Lys159, Lys171 and Lys196). A UCP mutant in which Cys118 was changed to alanine (UCP^C118A^) did not form a polyubiquitin chain but did strongly accumulate mono- and di-ubiquitin via auto-ubiquitination. Polyubiquitin chain formation required the coordination of Cys95 and Cys118 between two interacting molecules. The mechanism of the polyubiquitin chain reaction of UCP may involve the transfer of ubiquitin from Cys95 to Cys118 by trans-thiolation, with polyubiquitin chains forming at Cys118 by reversible thioester bonding. The polyubiquitin chains are then moved to the lysine residues of the substrate by irreversible isopeptide bonding. During the elongation of the ubiquitin chain, an active Cys118 residue is required in both parts of UCP, namely, the catalytic enzyme and the substrate. In conclusion, UCP possesses not only E2 ubiquitin conjugating enzyme activity but also E3 ubiquitin ligase activity, and Cys118 is critical for polyubiquitin chain formation.

## Introduction

Ubiquitin-mediated proteolysis is a powerful tool that eliminates unnecessary proteins in cells. The ubiquitination process requires three enzymes: E1, E2 and E3. The ubiquitin-activating enzyme E1 first forms a thioester bond between the active cysteine residue of E1 and the C-terminus of ubiquitin in an ATP-dependent manner [1]. Next, ubiquitin is transferred from E1 to the active cysteine residue of an E2 ubiquitin conjugating enzyme via trans-thiolation [2]. The final step of the ubiquitination process requires the E3 ubiquitin ligase, which recognizes the specific substrate and produces an isopeptide bond between a carboxyl group in ubiquitin and a lysine residue in the substrate protein [3]. Polyubiquitination can be assembled by the E3 via seven different lysine residues in ubiquitin, and each linkage has different structures and roles in cells. Most Lys48 linkages are involved in 26S proteasome-dependent proteolysis, whereas certain ubiquitin chains can lead to non-proteasomal signaling. For example, Lys63 linkages allow substrate proteins to participate in DNA repair, inflammation and other processes [4].

E2 enzymes consists of a highly conserved core domain containing an active cysteine residue. Although E2s share a common core domain, E2s have different enzymatic properties due to their unique C-terminal extensions. Certain E2s, such as CDC34, E2-25K and E2-EPF UCP (UCP), can directly transfer ubiquitin, self-associate into dimers or multimers and ubiquitinate themselves or specific substrates [5–7].

UCP has an active UBC domain that includes two cysteine residues: Cys95 and Cys118. UCP also has a unique basic C-terminal extension. This protein has been implicated in tumorigenesis and regulation of the cell cycle [8–11]. UCP can degrade von Hippel-Lindau protein (pVHL) via 26S proteasome-dependent proteolysis [9]. Ubiquitination of pVHL results in the stabilization of hydroxylated hypoxia-inducible factor (HIF)-1α, which induces the transcription of many hypoxia-inducible genes [12–14]. In cell cycle regulation, UCP works as an E2 enzyme within complexes containing E3, such as the anaphase-promoting complex (APC/C) [15]. In particular, UCP promotes the elongation of ubiquitin on a target substrate by the APC/C complex for proteasomal degradation [8].

In this study, we demonstrated the E3-independent polyubiquitination activity of UCP. The substrate recognition site for self-ubiquitination or pVHL ubiquitination was also discovered. Additionally, we suggest that the elongation of polyubiquitin chains by UCP occurred in *trans* and that Cys118 is the most critical site for building ubiquitin chains on the lysine residues of the substrate.

## Materials and Methods

### Plasmids

We generated plasmids encoding various truncated mutants of UCP, pVHL and wild-type UbcH5c for expression in bacterial cells or mammalian cells [9]. For bacterial expression, plasmids were constructed by ligating PCR products into pET28a (Novagen, WI, USA) and pGEX-4T-1 (GE Healthcare Life Sciences, WI, USA), and for mammalian expression, pFlag-CMV1 (Sigma-Aldrich, MO, USA), pEBG and pcDNA3-HA (Invitrogen, CA, USA) were used. Mutants of UCP and VHL were generated based on the wild-type genes using a PCR method [16].

### Recombinant protein extraction

All proteins were tagged with 6X His and expressed in *E*. *coli* BL21 (DE3). Cells harboring His-tagged protein expression plasmids were induced using IPTG (1 mM) at 37°C for 4 h. The induced cells were then harvested by centrifugation, resuspended in lysis buffer (20 mM Tris-HCl, 300 mM NaCl, 10 mM imidazole, and 1 mM PMSF, pH 7.5) and lysed by sonication on ice. The lysates were cleared by centrifugation, and the supernatants, containing the His-tagged proteins, were incubated with Ni-NTA agarose (Qiagen, Hilden, Germany) for 1 h at 4°C. The bead-protein complexes were loaded on a column and washed with washing buffer (20 mM Tris-HCl, 300 mM NaCl, and 20 mM imidazole, pH 7.5). The washed beads were subsequently eluted in elution buffer (20 mM Tris-HCl and 250 mM imidazole, pH 7.5), and the eluted proteins were dialyzed in dialysis buffer (10 mM Tris-HCl, 50 mM NaCl, 10% glycerol, 0.5 mM DTT, and 1 mM PMSF, pH 8.0) at 4°C overnight. The purified proteins were then dissolved in SDS sample buffer and separated by SDS-PAGE to analyze their concentration and purity. To purify GST-tagged recombinant proteins, cells were lysed in lysis buffer (137 mM NaCl, 2.7 mM KCl, 10 mM Na_2_HPO_4_, 2 mM KH_2_PO_4_ and 1 mM PMSF, pH 7.4) by sonication. The GST-tagged proteins were then purified from the cleared lysates by affinity purification using Glutathione Sepharose beads (GE Healthcare Life Sciences).

### Cell culture and transient transfection

HEK-293T and HeLa cells were cultured at 37°C in a humidified 5% CO_2_ atmosphere in Dulbecco’s modified Eagle’s medium (DMEM) supplemented with 10% (v/v) fetal bovine serum (FBS) (Gibco, NY, USA) and 1X (v/v) antibiotics (Gibco). HEK-293T cells (3 X 10^6^ cells/10 cm dish) were transiently transfected using the standard calcium-phosphate method. To identify ubiquitination in the cells, we incubated transiently transfected cells in the presence of 10 μM MG132 (Sigma-Aldrich) for 12 h before harvesting for analysis at 48 h post-transfection.

### shRNA construct and stable-knockdown cell line

A UCP gene-specific shRNA construct and shRNA-control were synthesized using shRNA-UCP (5'-GAAGCTGGCGGCCAAGAAA-3') and shRNA-control (5'-AAGGAGACGAGCAAGAGAA-3') oligonucleotides that were cloned into the pSuper vector (Oligoengine, WA, USA). HeLa cells (3 X 10^5^ cells/well) were plated on 6-well plates and co-transfected with 2.5 μg/well of UCP specific shRNA plasmid and 2.5 μg/well of pTK plasmid using Lipofectamine 2000 (Invitrogen). Following transfection, the medium was replaced with DMEM containing 500 μg/ml hygromycin B (Invitrogen) for selection. To determine the UCP knockdown efficiency, the cultured cells were lysed in RIPA buffer (10 mM Tris-HCl, 150 mM NaCl, 1% NP-40, 1% sodium deoxycholate, 0.1% SDS, and 0.02% sodium azide, pH 8.0), and the lysates were analyzed by immunoblotting using a UCP-specific antibody.

### Antibodies and immunoblotting

We purchased primary antibodies against Flag (Sigma-Aldrich), His (Santa Cruz Biotechnology), HA (AbFrontier, Seoul, Korea) and β-actin (Sigma-Aldrich). Horseradish peroxidase (HRP)-conjugated anti-mouse and anti-rabbit IgG (Santa Cruz Biotechnology) were used as secondary antibodies. We also purchased anti-ubiquitin antibodies from Santa Cruz Biotechnology (Cat. # sc-8017) and Cell Signaling Technology (Cat. # 3933) to compare the patterns of ubiquitination. The antibody purchased from Santa Cruz Biotechnology was mainly used in this research. In addition, we generated UCP- and GST-specific antibodies by immunizing mice with His-UCP and GST, respectively. For immunoblotting, cells were lysed in RIPA buffer, and proteins were mixed in 2X SDS sample buffer and analyzed using specific antibodies.

### *In vitro and in vivo* ubiquitination assay

For the *in vitro* ubiquitination assay, reaction mixtures were incubated with 0.5 μg of His-E1, 0.2 μg of UCP, and 1.25 μg of Flag-ubiquitin (Sigma-Aldrich) in reaction buffer (25 mM Tris-HCl pH 7.5, 1 mM MgCl_2_, 2.5 mM DTT, 5 mM ATP, and ATP regeneration system [1 mM creatine phosphate (Sigma-Aldrich), 1 mM creatine kinase (Sigma-Aldrich), and 0.5 μg/ml ubiquitin aldehyde (Boston Biochem, MA, USA)]) at 37°C for 1 h, and the reactions were stopped by boiling in 2X SDS sample buffer. The samples were then separated by SDS-PAGE under denaturing (10% β-mercaptoethanol) or non-denaturing (0% β-mercaptoethanol) conditions and visualized by immunoblotting. The ubiquitinated proteins were mainly separated under denaturing conditions. To identify the linkage-specific ubiquitin chain formation resulting from UCP auto-ubiquitination, we used lysine-to-arginine ubiquitin mutants (K6R, K11R, K48R and K63R; Boston Biochem), single-lysine ubiquitin mutants (K6, K11, K48 and K63; Boston Biochem) and a lysine-null ubiquitin mutant (K-null; Boston Biochem). For the *in vitro* pVHL ubiquitination assay, reaction mixtures were incubated 0.5 μg of His-E1, 0.2 μg of UCP, 2 μg of pVHL and 1.25 μg Flag-ubiquitin in reaction buffer along with the ATP regeneration system at 37°C for 1 h. To precipitate the ubiquitinated pVHL, the total reaction mixtures were pulled down with Glutathione Sepharose beads or Ni-NTA agarose on a rotary shaker at 4°C for 3 h. The beads were then washed three times under reducing (1% NP40 in NET gel buffer) or non-reducing (4 M urea and 1% NP40 in NET gel buffer) conditions and resuspended in 2X SDS sample buffer under denaturing conditions. For the *in vivo* ubiquitination assay, cells were co-transfected with indicated plasmids and treated with 10 μM MG132 for 12 h before harvesting. A cell lysates were subsequently prepared in NET gel buffer (50 mM Tris-HCl pH 7.5, 150 mM NaCl, 0.1% NP-40, and 1 mM EDTA, pH 8.0) supplemented with complete proteinase inhibitor cocktail (Roche, Basel, Switzerland), and pulled down with Glutathione Sepharose beads. The ubiquitinated proteins were then separated by SDS-PAGE and analyzed by immunoblotting.

## Results

### UCP ubiquitinates itself independent of E3 ubiquitin ligase *in vitro* using mixed lysine linkages

**Fig 1:**
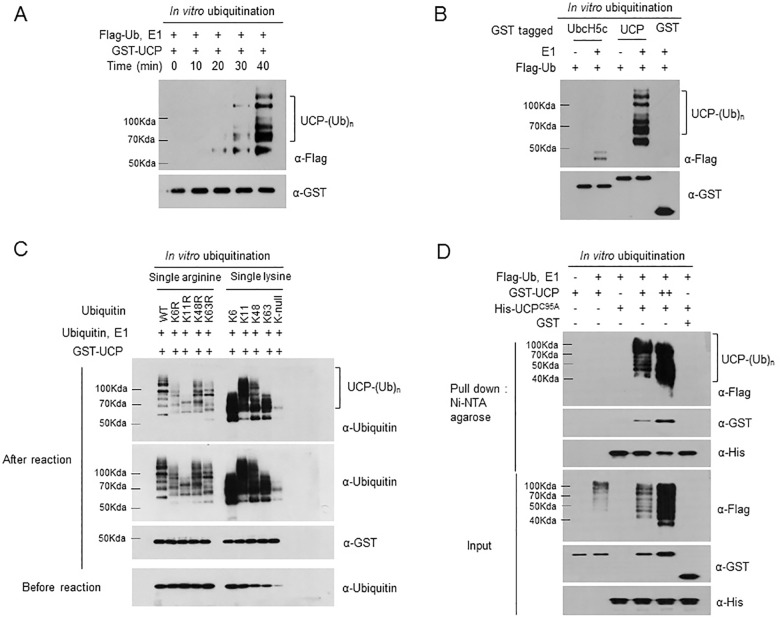
Autoubiquitination of UCP Occurs in an E3-independent Manner. (A) An autoubiquitination assay was performed using UCP at various time points. GST-UCP (0.2 μg) was incubated at 37°C in the presence of E1 and Flag-ubiquitin, and autoubiquitination was visualized with anti-Flag antibody. (B) The catalytic ability of UncH5c proteins was analyzed in an *in vitro* autoubiquitination assay. GST-UbcH5c proteins (0.2 μg) and GST-UCP (0.2 μg) were incubated at 37°C for 40 min in the presence of E1 and Flag-ubiquitin, and the ubiquitinated proteins were detected by immunoblotting using anti-Flag antibody. (C) The lysine-specific linkage of UCP was defined using lysine-to-arginine ubiquitin mutants (K6R, K11R, K48R and K63R), single-lysine ubiquitin mutants (K6, K11, K48 and K63) and a lysine-null ubiquitin mutant (K-null). Autoubiquitination assays were performed using GST-UCP (0.2 μg) and wild type ubiquitin or ubiquitin mutants at 37°C for 1 h, and ubiquitinated proteins were detected by immunoblotting using anti-ubiquitin antibody. (D) GST-UCP (0.2 μg) and His-UCP^C95A^ (2 μg) were incubated at 37°C for 1 h in the presence of E1 and Flag-ubiquitin. His-UCP^C95A^ was then pulled down with Ni-NTA agarose, and His-UCP^C95A^ polyubiquitination was detected by immunoblotting using anti-Flag antibody.

In previous studies, we found that UCP ubiquitinates both pVHL and itself [9]. Here, we investigated the detailed biochemical conditions required for the polyubiquitination activity of the UCP protein. We first found that self-ubiquitination of UCP proceeded in a time-dependent manner (Fig 1A). However, another typical E2, UbcH5c, and the control protein, GST, did not exhibit polyubiquitin chain accumulation in autoubiquitination assay (Fig 1B). To define the specific lysine linkage type in UCP autoubiquitination, UCP autoubiquitination was observed in the presence of wild-type ubiquitin and lysine-to-arginine mutants (K6R, K11R, K48R and K63R) *in vitro* (Fig 1C, left panel). UCP did not initiate chain elongation using the K11R ubiquitin mutant. Additionally, we observed that the accumulation of the polyubiquitin chains was decreased using the K6R, K48R and K63R ubiquitin mutants compared with wild-type ubiquitin. To confirm this result, we carried out UCP autoubiquitination using single-lysine ubiquitin mutants (K-6-only, K-11-only, K48-only and K63-only) and a lysine-null ubiquitin mutant (K-null) and found that UCP could not use the single-lysine ubiquitin mutants to form polyubiquitin chains (Fig 1C, right panel). UCP used all of the single-lysine ubiquitin mutants for autoubiquitination, and it used the K-11-only ubiquitin mutant most efficiently. This result suggested that UCP autoubiquitination includes K6, K48 and K63 linkages in addition to K11 linkages. Additionally, we observed that the patterns of UCP autoubiquitination were dependent on the antibody used (S1 Fig). Specifically, the antibody from Cell Signaling Technology (Cat. # 3933) did not recognize several linkages (K6R, K11R, K11-only, K48-only and K63-only), whereas the antibody purchased from Santa Cruz Biotechnology (# 8017) recognized ubiquitin chains of all linkages; therefore, we used the latter antibody for further experiments. To verify whether the autoubiquitination of UCP occurs *in trans*, autoubiquitination assays were performed using UCP-C95A (UCP^C95A^), which is a catalytically inactive mutant, as the substrate. At 1 h post-reaction, His-UCP^C95A^ proteins were selectively pulled down from the total reaction mixture using Ni-NTA agarose, and then the ubiquitination was analyzed by immunoblotting. The data showed that wild-type UCP was able to ubiquitinate His-UCP^C95A^, indicating that the autoubiquitination of UCP occurs *in trans* (Fig 1D). Taken together, these observations suggested that UCP formed polyubiquitin chains independently of an E3 ubiquitin ligase *in trans* manner.

### The UBC domain of UCP is the critical region for substrate recognition

**Fig 2:**
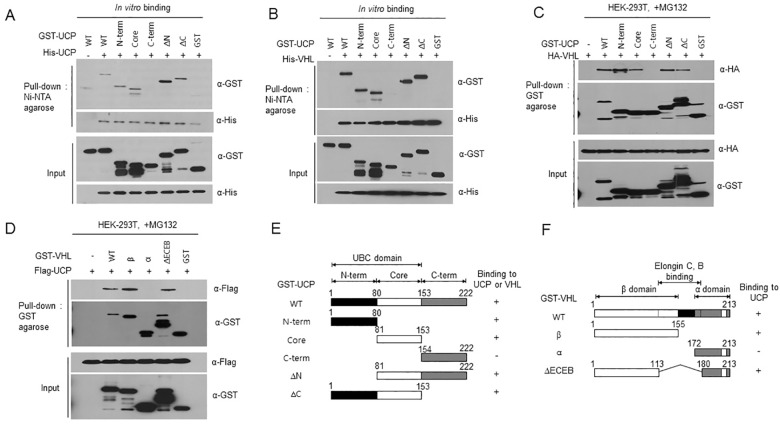
The N-terminal and Core Domains of UCP are Critical for Substrate Binding. (A) His-UCP^WT^ (1 μg) and GST-UCP^WT^ (1 μg) or truncated UCP mutants (UCP^N-term^, UCP^Core^, UCP^C-term^, UCP^∆N^ and UCP^∆C^) (1 μg) were incubated at 4°C for 2 h. His-UCP was then pulled down with Ni-NTA agarose at 4°C for 2 h, and the bound domains were detected by immunoblotting. (B) His-VHL (1 μg) and GST-UCP^WT^ (1 μg) or truncated UCP mutants (1 μg) were incubated at 4°C for 2 h. His-UCP was then pulled down with Ni-NTA agarose, and the bound domains were detected by immunoblotting. (C) HA-VHL (5 μg) and GST-UCP^WT^ (5 μg) or truncated UCP mutant plasmids (5 μg, each) were co-transfected into HEK-293T cells. GST-UCP proteins were pulled down with GST-resin, and then HA-VHL was detected by immunoblotting. (D) The plasmids Flag-UCP^WT^ (5 μg) and GST-VHL^WT^ or truncated VHL mutants (β, α, ∆ECEB) (5 μg, each) were co-transfected into HEK-293T cells. GST-VHL was pulled down with GST-agarose, and then interaction with UCP was detected by immunoblotting using anti-Flag antibody. (E, F) Schematic representation of UCP and pVHL. The functional domains of UCP and pVHL are delineated by three colored boxes (white, gray and black). The ability of the different domains to bind UCP and VHL is indicated; +; binding; -; no binding.

E2 proteins have a UBC domain, a conserved region that consists of approximately 150 amino acids containing catalytically active cysteine residue. The UCP protein in particular comprises 222 amino acids harboring a UBC domain (1–153), whereas no HECT or RING domain is present. To identify the critical region for polyubiquitination of UCP itself and pVHL, we constructed truncated mutants of UCP (N-terminus, core, C-terminus, ∆N and ∆C). To identify the substrate recognition site for autoubiquitination and for pVHL polyubiquitination, an *in vitro* binding assay was performed using His-UCP (wild-type), pVHL (wild-type) and GST-UCP mutants (UCP^N-term^, UCP^Core^, UCP^C-term^, UCP^∆N^ and UCP^∆C^). The results showed that regions of the N-terminus, core, ∆N and ∆C domains are involved in UCP’s interaction with itself and in binding pVHL, while the C-terminal region is not (Fig 2A and 2B). As UCP and pVHL share common interaction sites, they probably compete with each other. To confirm the UCP-binding region on pVHL *in vivo*, we co-transfected the plasmids encoding HA-VHL and GST-UCP mutants (UCP^N-term^, UCP^Core^, UCP^C-term^, UCP^∆N^ and UCP^∆C^) into HEK-293T cells and obtained the same results as *in vitro* (Fig 2C). To further identity the binding domains of pVHL, we constructed truncated pVHL mutants (VHL^β^, VHL^α^, and UCP^∆ECEB^) and we co-transfected wild-type UCP (Flag-tagged UCP) and pVHL mutants into HEK-293T cells (Fig 2D). The schematic illustrations of truncated UCP and pVHL mutants were represented in Fig 2E and 2F. The results revealed that the β-domain of pVHL interacts with UCP. Therefore, the UBC domain region of UCP is critical for UCP’s interaction with substrate proteins.

### The UBC domain of UCP can form polyubiquitin chains on UCP itself and pVHL

**Fig 3:**
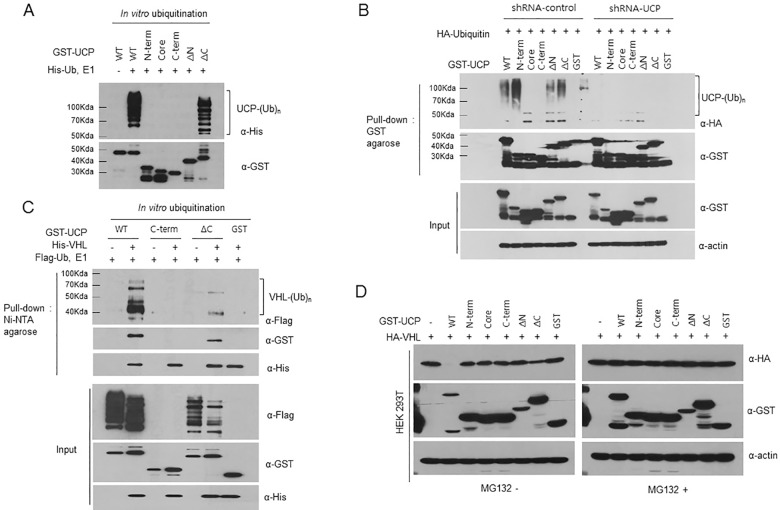
The UBC Domain of UCP can Forms Polyubiquitin Chains on the N-terminus. (A) The catalytic activity of UCP^WT^ (0.2 μg) and truncated UCP mutants (UCP^N-term^, UCP^Core^, UCP^C-term^, UCP^∆N^ and UCP^∆C^) (0.2 μg) was assessed using an *in vitro* ubiquitination assay. The proteins were incubated at 37°C for 1 h in the presence of E1 and His-ubiquitin, and the ubiquitinated proteins were detected by immunoblotting using anti-His antibody. (B) The substrate region of UCP for autoubiquitination was analyzed in stable HeLa cell lines transfected with shRNA-control or shRNA-UCP. Different stable HeLa cell lines were co-transfected with plasmids encoding each truncated UCP mutant (5 μg) and HA-Ubiquitin (2 μg), treated with 10μM MG132 for 12 h and harvested at 48 h post-transfection. The cells were then lysed, and UCP was pulled down with GST-agarose. The ubiquitinated domains were subsequently detected by immunoblotting using anti-HA antibody. (C) GST-tagged UCP^WT^ (0.2 μg) and truncated UCP mutants (UCP^C-term^ and UCP^∆C^, 0.2 μg) were mixed with His-VHL (2 μg) and subjected to an *in vitro* ubiquitination assays at 37°C for 1 h. pVHL was pulled down with Ni-NTA agarose, and ubiquitinated forms were detected by immunoblotting using anti-Flag antibody. (D) HA-VHL (5 μg) and GST-wild-type UCP or truncated UCP mutant plasmids (5 μg) were co-transfected into HEK-293T cells and treated with/without 10 μM MG132 for 12 h. Changes in the pVHL expression level were then detected by immunoblotting.

To test the catalytic activity of each domain of UCP, we performed an *in vitro* autoubiquitination assay using UCP mutants (UCP^N-term^, UCP^Core^, UCP^C-term^, UCP^∆N^ and UCP^∆C^). The partial UBC domains (UCP^N-term^, UCP^Core^, UCP^C-term^, and UCP^∆N^) were unable to extend ubiquitin chains. By contrast, the C-terminal deletion mutant (UCP^∆C^) had catalytic activity, but this activity was weaker than that of wild-type UCP (UCP^WT^) (Fig 3A). In a previous experiment, we found that the N-terminus of UCP (UCP^N-term^) was strongly ubiquitinated *in vivo*, as opposed to the data in Fig 3A (S2 Fig), and we hypothesized that endogenous UCP elongates polyubiquitin chains on its N-terminus. To test the catalytic activity of endogenous UCP *in vivo*, we produced a stable HeLa cell line expressing UCP shRNA, which depleted endogenous UCP (S3 Fig). The shRNA-UCP expressing or shRNA-control cell lines were transfected with GST-UCP (UCP^WT^), the N-terminus of UCP (UCP^N-term^) and other UCP mutants (UCP^Core^, UCP^C-term^, UCP^∆N^ and UCP^∆C^) (Fig 3B). The data showed that UCP^WT^ and UCP^N-term^ were strongly ubiquitinated in the presence of endogenous UCP (Fig 3B, left panel) but were not ubiquitinated in the absence of endogenous UCP (Fig 3B, right panel). This result suggested that the UBC domain plays a critical role in autoubiquitination and that the N-terminal region of UCP is most strongly modified *in vivo*. Because the N-terminus does not have catalytic activity, it was hypothesized that lysines serving as sites for the ligation of polyubiquitin chains exist in the N-terminus of UCP. Additionally, we tested whether UCP^WT^ and UCP^∆C^, which are catalytically active in autoubiquitination, also elongated polyubiquitin chains on pVHL (Fig 3C). UCP^WT^ ubiquitinated pVHL sufficiently, and UCP^∆C^ exhibited weaker polyubiquitin chain formation than UCP^WT^ did. This result implied that autoubiquitination and pVHL ubiquitination are linked not only by interaction but also by catalytic activity. To distinguish between UCP autoubiquitination and polyubiquitination of pVHL, we performed a pull-down assay using Ni-NTA agarose under non-reducing or reducing conditions and then visualized the ubiquitinated proteins by immunoblotting (S4 Fig). To confirm that the polyubiquitination activity corresponded to pVHL degradation *in vivo*, HA-VHL and GST-UCP mutants (UCP^N-term^, UCP^Core^, UCP^C-term^, UCP^∆N^ and UCP^∆C^) were co-transfected into HEK-293T cells (Fig 3D). Wild-type UCP and the C-terminal deletion UCP mutant (UCP^∆C^) induced a decreasing protein level of pVHL *in vivo*, suggesting that the UBC domain is a catalytically important region and that pVHL polyubiquitination by wild type UCP or UCP^∆C^ causes its proteasomal degradation.

### UCP elongates the polyubiquitin chain on the lysine residues of its substrate

**Fig 4:**
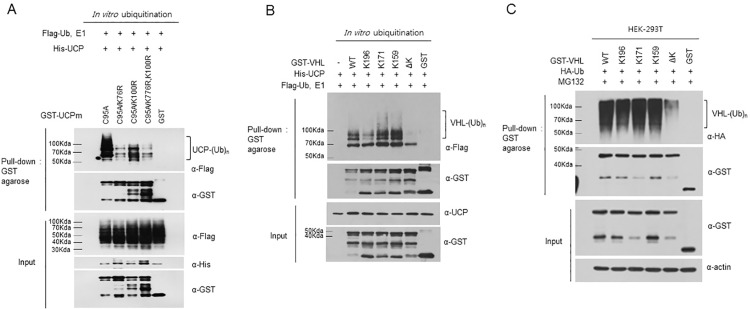
UCP Forms Polyubiquitin Chains on Specific Lysine Residues in its Substrate. (A) *In vitro* ubiquitination assays were performed using His-UCP^WT^ as the enzyme and inactive UCP mutants containing lysine-to-arginine mutations as the substrates (GST-UCP-C95A, GST-UCP- C95A/K76R, GST-UCP-C95A/K100R, and GST-UCP-C95A/K76R,K100R) (2 μg). After the reaction, GST-UCP was pulled down with GST agarose at 4°C for 2 h. The ubiquitinated forms were visualized with anti-Flag antibody. (B) An *in vitro* ubiquitination assay was performed using His-UCP^WT^ (0.2 μg) and wild-type and/or single-lysine pVHL mutants (VHL^K159^, VHL^K171^, VHL^K196^ and VHL^∆K^) (2 μg) at 37°C for 1 h. After incubation, pVHL was pulled down with GST agarose, and ubiquitinated forms were detected by immunoblotting using anti-Flag antibody. (C) Wild-type pVHL (5 μg), single-lysine pVHL (5 μg) mutant and HA-Ubiquitin plasmids (2 μg) were co-transfected into HEK-293T cells, which were then incubated with 10 μM MG132 for 12 h. After the cells were lysed, pVHL was pulled down with GST agarose, and ubiquitinated forms were detected by immunoblotting with anti-HA antibody.

According to the results described above, the UBC domain of UCP is the catalytic domain for autoubiquitination and pVHL ubiquitination. To determine the ubiquitination site of the substrate in autoubiquitination and pVHL polyubiquitination, we constructed UCP and pVHL mutants bearing altered lysines; the lysine residues in the UBC domain of UCP and pVHL are illustrated in S5 Fig. To verify which lysine residue plays a role in autoubiquitination, we constructed UCP mutants, with one lysine residue changed to arginine (K18R, K32R, K63R, K68R, K76R, K82R, K100R or K117R). *In vitro* autoubiquitination assays were then performed using these lysine mutants. The data revealed that the polyubiquitin chain extension ability of the UCP-K76R mutant (UCP^K76R^) was significantly weaker than that of wild-type UCP (S6 Fig). Similarly, the UCP-K100R mutant (UCP^K100R^) showed slightly shorter ubiquitin chain elongation (S6 Fig). UCP recognizes itself as a substrate and elongates polyubiquitin chains on target lysine residues. To determine whether UCP elongates polyubiquitin chains specifically at Lys76 or Lys100, we generated UCP mutants containing a lysine-to-arginine mutation at position 76 or 100, as well as a double mutant, which both Lys76 and 100 mutated to arginine. UCP operates as both the enzyme and the substrate. Cys95 of UCP is an essential catalytic residue; thus, the cysteine 95-to-alanine mutant affects only the substrate. Each of the three arginine mutants were incorporated into double or triple UCP mutants in which the catalytic site Cys95 was mutated to alanine for use as the substrate in *in vitro* ubiquitination assays. The UCP-C95A-K76R or UCP-C95A-K76R/K100R mutants were significantly weaker substrates than wild-type UCP, whereas UCP-C95A and UCP-C95A-K100R mutants showed no change. The results showed that UCP ubiquitinates specific lysines (Fig 4A). Similarly, we determined whether UCP has lysine specificity in pVHL ubiquitination both *in vitro* and *in vivo*. pVHL contains three lysine residues (K159, K171 and K196); therefore, we produced pVHL mutants containing only a single lysine, with the other lysines mutated to arginine (VHL^K159^, VHL^K171^, and VHL^K196^), as well as a lysine-null mutant (VHL^∆K^). The lysine-null pVHL mutant could not be ubiquitinated by UCP in an *in vitro* ubiquitination assay (Fig 4B), and the polyubiquitination of VHL^K196^ was decreased. To perform an *in vivo* ubiquitination assay, plasmids encoding each of the lysine mutants (VHL^K159^, VHL^K171^, VHL^K196^ and VHL^∆K^) were co-transfected into HEK-293T cells along with an HA-Ubiquitin plasmid and treated with MG132 for 12 h. The differences between the pVHL lysine mutants were not significant. The results showed that UCP elongates polyubiquitin chains on all lysine residues in pVHL. Therefore, we concluded that UCP recognizes its substrate via a specific interaction and then performs polyubiquitination on the lysine residues of its substrate (Fig 4C).

### Cys118 plays a critical role in polyubiquitin chain formation by UCP

**Fig 5:**
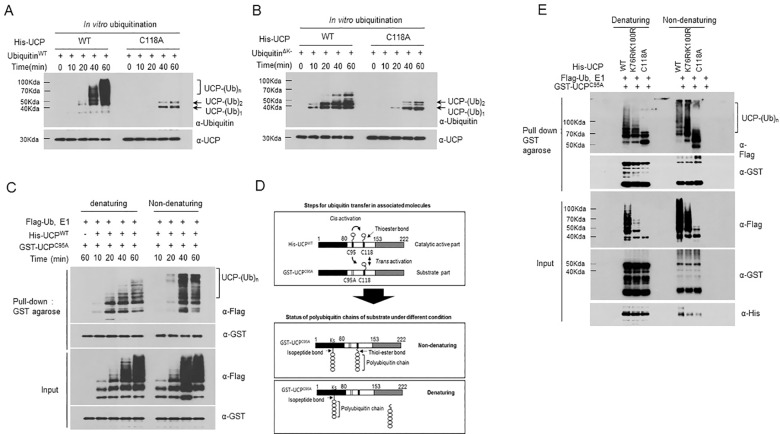
The Residue Cys118 Plays a Key Role in the Autoubiquitination of UCP. (A) An autoubiquitination assay was performed using His-UCP^WT^ and His-UCP^C118A^ (0.2 μg each) along with wild-type ubiquitin (Ub^WT^) at various time points. Ubiquitinated forms were detected by immunoblotting using anti-ubiquitin antibody. (B) An autoubiquitination assay was performed using His-UCP^WT^ and His-UCP^C118A^ (0.2 μg each) along with lysine-null ubiquitin (Ub^∆K^) at various time points. Ubiquitinated forms were detected by immunoblotting using anti-ubiquitin antibody. (C) *In vitro* ubiquitination assay was performed using His-UCP^WT^ (0.2 μg) and GST-UCP^C95A^ (2 μg) at various time points. GST-UCP^C95A^ was pulled down using GST agarose and then polyubiquitination was separated by SDS-PAGE under denaturing or non-denaturing (without β-mercaptoethanol) condition. The polyubiquitin chains were detected by anti-Flag antibody. (D) Illustration of the expected reaction steps for polyubiquitination by UCP in the *trans* manner and the status of polyubiquitin chains on the substrate under different conditions. (E) An *in vitro* ubiquitination assay was performed using wild-type UCP (His-UCP^WT^), double-mutant UCP with K78R and K100R (His-UCP^K76R/K100R^), UCP-C118A mutant (His-UCP^C118A^) (0.2 μg each) and GST-UCP^C95A^ (2 μg) at 37°C for 1 h in reaction buffer. After the reaction, GST-UCP^C95A^ was pulled down with GST agarose, and polyubiquitination was analyzed by immunoblotting under denaturing (+β-mercaptoethanol) or non-denaturing (-β-mercaptoethanol) conditions using anti-Flag antibody.

UCP has a Cys118 residue in addition to a Cys95 residue in its UBC domain. Cys95 clearly initiates ubiquitin charging, but this does not fully explain polyubiquitination by UCP. These data thus led us to focus on the role of the Cys118 residue in polyubiquitination. To demonstrate the role of Cys118 in UCP, we constructed a UCP mutant in which Cys118 was changed to alanine (UCP^C118A^), and then we examined autoubiquitination using UCP^C118A^ *in vitro*. UCP^WT^ showed polyubiquitin chains in a time-dependent manner, whereas the UCP^C118A^ mutant accumulated mono-ubiquitin and di-ubiquitin at its end (Fig 5A). To verify whether the UCP^C118A^ mutant formed a mono- or di-ubiquitin chain, we performed an autoubiquitination assay using a lysine-null ubiquitin mutant (Ub^∆K^), which was not able to be polyubiquitinated. As expected, neither UCP^WT^ nor UCP^C118A^ could form polyubiquitin chains, but both showed a mono- or di-ubiquitin chain (Fig 5B). These data suggested that the Cys118 residue is involved in forming polyubiquitin chains during UCP self-ubiquitination. As shown in Fig 1D, autoubiquitination of UCP occurred in a *trans* manner, which means that one molecule acts as the enzyme and the other acts as the substrate. Previous reports have shown that a cysteine in E2 is linked to polyubiquitin chains by a thioester bond [17]. To examine this possibility, we performed an *in vitro* ubiquitination assay using UCP^WT^ as the enzyme and UCP^C95A^ as the substrate. Polyubiquitin chains were observed on UCP^C95A^ under both denaturing and non-denaturing conditions. Both thiolation and isopeptide bonds were revealed under non-denaturing conditions, whereas only isopeptide bonds were detected under denaturing conditions (Fig 5C). Thioesterification to cysteine and isopeptide bonds to lysine are involved in the autoubiquitination process. To distinguish between thioesterification and isopeptide bonds, we performed an *in vitro* UCP^C95A^ polyubiquitination assay using wild-type UCP (UCP^WT^), double-mutant UCP containing K76R and K100R (UCP^K76R/K100R^) and C118A-mutant UCP (UCP^C118A^) as catalysts because they have different catalytic activities, and the analysis was performed under both denaturing and non-denaturing conditions. UCP^WT^ and UCP^K76R/K100R^ showed polyubiquitin chains under denaturing and non-denaturing conditions, but they produced higher-molecular-weight polyubiquitin chains under non-denaturing conditions. Because UCP^K76R/K100R^ possesses weaker isopeptide bond formation than UCP^WT^, the former showed relatively weak polyubiquitination patterns under denaturing conditions. In contrast, UCP^C118A^, which has defective thioesterase activity, revealed mono- and di-ubiquitination under both conditions. These results indicate that Cys118 is involved in both trans-thiolation and polyubiquitination on substrates (Fig 5E). We suggest that ubiquitin transfer (trans-thiolation) occurred between Cys95 and Cys118 both in the *trans* and *cis* manners and that the elongated ubiquitin chain was then transferred to the substrate via Cys118 only in the *trans* manner (Fig 5D).

### All active Cys118 residues in a UCP complex are essential for polyubiquitin chain formation

**Fig 6:**
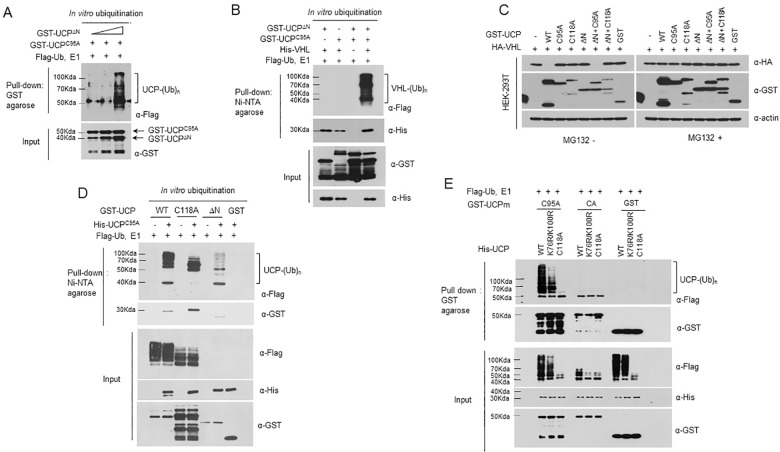
Intermolecular Active Cys118 Residues are Required for Autoubiquitination of UCP. (A) Autoubiquitination was assessed using GST-UCP^C95A^ (1 μg) and GST-UCP^∆N^ (0.5, 1, or 2 μg), and polyubiquitination was analyzed by immunoblotting using anti-Flag antibody. (B) An *in vitro* ubiquitination assay was performed using the protein pair GST-UCP^∆N^/GST-UCP^C95A^ (each 0.2 μg) and His-VHL (2 μg). His-VHL protein was pulled down with Ni-NTA agarose, and ubiquitinated forms were analyzed by immunoblotting using anti-Flag antibody. (C) HA-VHL and UCP^WT^ or UCP mutants (UCP^C95A^, UCP^C118A^ and UCP^∆N^) or various pairs of UCP mutant (UCP^C95A^/UCP^ΔN^, UCP^ΔN^/UCP^C118A^) plasmids (total of 10 μg) were co-transfected into HEK-293T cells and treated with/without 10 μM MG132 for 12 h. At 48 h post-transfection, the cells were harvested and lysed. Changes in pVHL expression levels were detected by immunoblotting. (D) An *in vitro* ubiquitination assay was performed using wild-type UCP (0.2 μg) or UCP mutants (GST-UCP^C118A^ and GST-UCP^∆N^, each 0.2 μg) and His-UCP^C95A^ (2 μg). His-UCP^C95A^ protein was pulled down with Ni-NTA agarose, and ubiquitinated forms of His-UCP^C95A^ were assessed by immunoblotting using anti-Flag antibody. (E) An *in vitro* ubiquitination assay was performed using wild-type UCP (0.2 μg), double-mutant UCP with K76R and K100R (His-UCP^K76R/K100R,^ 0.2 μg), C118A-mutant UCP (His-UCP^C118A,^ 0.2 μg) and GST-UCP^C95A^ or GST-UCP^CA^ (2 μg) at 37°C for 1 h. The GST-UCP^C95A^ or GST-UCP^CA^ protein was pulled down with GST agarose and analyzed by immunoblotting using anti-Flag antibody.

UCP autoubiquitination requires coordination between the catalytic UBC domain and polyubiquitin chain elongation sites. Because UCP ubiquitinates itself with recognition in the *trans* manner, we hypothesized that the damaged activity of Cys95A can be complemented by active Cys95 in a paired molecule. To address this issue, we performed an *in vitro* ubiquitination assay using the pair UCP^C95A^/UCP^ΔN^, in which polyubiquitin chain elongation occurred at the lysine residue of UCP^C95A^ (Fig 6A). In this context, we examined whether the complex could ubiquitinate pVHL *in vitro* and found that the UCP^C95A^/UCP^ΔN^ pair polyubiquitinated pVHL coordinately (Fig 6B). Although UCP^ΔN^ harbored catalytic active cysteine residues, it could not ubiquitinate pVHL due to the missing N-terminus, which forms part of the binding site for pVHL. In addition, we determined whether the polyubiquitination of pVHL by the UCP^C95A^/UCP^ΔN^ pair could induce proteasomal degradation and found that the UCP^C95A^/UCP^ΔN^ pair degraded pVHL *in vivo*, whereas the UCP^ΔN^/UCP^C118A^ pair could not degrade pVHL (Fig 6C). These results indicated that the catalytic activity of Cys95 can be complemented by a paired molecule. To verify this concept, we carried out an autoubiquitination assay using various UCP mutants (UCP^WT^, UCP^C118A^ and UCP^ΔN^) as the catalyst and UCP^C95A^ as the substrate (Fig 6D). The results suggested that UCP^ΔN^ formed polyubiquitin chains on the N-terminus of UCP^C95A^, whereas UCP^C118A^ accumulated mono- and di-ubiquitin. To verify the role of Cys118, we constructed a UCP^CA^ mutant, in which both cysteine residues were changed to alanine. We then performed an *in vitro* ubiquitination assay using wild-type UCP (UCP^WT^), double-mutant UCP containing K76R and K100R (UCP^K76R/K100R^) and C118A-mutant UCP (UCP^C118A^) as the catalyst and UCP^C95A^ and UCP^CA^ as the substrates (Fig 6E). When Cys118 was active on both sides, autoubiquitination occurred, such as in the pairs UCP^WT^/UCP^C95A^ and UCP^K76R/K100R^/UCP^C95A^. However, the pair including UCP^CA^ did not form polyubiquitin chains. These data supported the idea that Cys118 cannot be complemented in UCP self-association; therefore, two active Cys118 residues in a UCP dimer are required for polyubiquitin chain formation. These data also led us to test the catalytic activity of the pair UCP^CA^/UCP^ΔN^. In particular, we directly compared the catalytic activity of two pairs, namely, UCP^C95A^/UCP^ΔN^ and UCP^CA^/UCP^ΔN^, using an autoubiquitination assay *in vitro*. We found that UCP^CA^/UCP^ΔN^ was not able to form a polyubiquitin chain but strongly accumulated mono-ubiquitin (S8A Fig). The difference between the two pairs was the status of Cys118. The expected processes of autoubiquitination are illustrated in S8B Fig. The data showed that the Cys118 residue is required to form polyubiquitin chains. Therefore, we conclude that UCP possesses E3 ubiquitin ligase activity and that the Cys118 residue plays a key role in polyubiquitination.

## Discussion

Autoubiquitination activity has been identified in certain types of E2 proteins, such as E2-25k, CDC34 and UCP [18, 19]. UCP is an oncoprotein that targets pVHL and can ubiquitinate it both *in vitro* and *in vivo*. UCP thus can degrade pVHL proteasome dependently and thereby promotes tumorigenesis [9]. The role and mechanism of UCP as an E2 ubiquitin conjugating enzyme are well established, but its catalytic activity as an E3 ubiquitin ligase is still being explored. Here, we identified the mechanism of how UCP forms polyubiquitin chains on itself and on pVHL without the help of another E3 ubiquitin ligase.

polyubiquitin chain assembly by UCP required not only K48 but also K6, K11 and K63 linkages for both autoubiquitination (Fig 1) and pVHL ubiquitination (data not shown). The K11 linkage specifically initiates chain elongation [10, 20, 21]. UCP initiates polyubiquitin chain elongation using K11 in the APC/C complex, which is a determining factor in polyubiquitin chain elongation [22]. These data provide evidence that UCP plays different roles under different molecular conditions. For example, for proteasomal degradation, UCP assembled a polyubiquitin chain on itself and on pVHL (data not shown) using K6, K11, K48 and K63 linkages. At the same time, UCP serves as an E2 ubiquitin conjugating enzyme that initiates ubiquitin chain elongation using the K11 linkage with members of the APC/C complex. However, which factor regulates the destination of UCP in cells is unknown.

Our results showed that the autoubiquitination of UCP occurs through an intermolecular association in a *trans* manner (Fig 2). Most E3 ubiquitin ligases catalyze their substrate by autoubiquitination, and certain E2 proteins have autoubiquitination-related catalytic activity [6, 7, 23]. In the autoubiquitination mechanism, the relationship between self-association and function is not clear, but several reports have observed that enzymatic function is dependent on self-association and the ability to ubiquitinate its substrate [24, 25]. The results presented here indicated that self-associates through the UBC domain. The UBC domain also contributed to the interaction with pVHL. We observed similar formation of polyubiquitin chains between UCP autoubiquitination and pVHL ubiquitination. These phenomena can be explained by the fact that the proteins share the same catalytic domain. Therefore, competition can occur between UCP and pVHL in the same region. If UCP interaction with pVHL is more frequent than interaction with itself, this could result in the loss of pVHL function, thereby promoting tumorigenesis. However, the factor that allows UCP to distinguish between itself and pVHL is not clear. Meanwhile, it is clear that the UBC domain of UCP is involved in both substrate recognition and catalytic activity (Fig 3). However, the UBC domain without its N-terminus (a.a. 1–80) could not form polyubiquitin chains. We thus speculated that the lysine residues of the N-terminal region of UCP can serve as the autoubiquitination site of UCP. Indeed, the N-terminal region was ubiquitinated by endogenous UCP *in vivo*. We defined specific lysine residues for autoubiquitination and pVHL ubiquitination (Fig 4). Regarding autoubiquitination, Lys76 was the most critical site, and Lys100 was also weakly involved in ubiquitination. Because Lys76 and Lys100 are near the catalytic active site (Cys95), these residues could be recognized more easily than other lysine residues (S7 Fig). The structural limitations on E3’s area of action provide lysine specificity for the substrate [26, 27]. We suggest that the UBC domain of UCP not only recognizes but also forms polyubiquitin chains on substrates, such as the HECT domain [28]. We also confirmed the lysine specificity of pVHL ubiquitination. To do so, we generated pVHL mutants that had a single lysine (K159, K171, and K196) or that were lysine null (∆K). We observed the formation of polyubiquitin chains at all three lysines in pVHL, and the results showed that polyubiquitination at Lys159 and Lys171 was more sensitive than ubiquitination a Lys196 in *in vitro* ubiquitination assays. However, the difference in intensity between three lysines was lower than the lysine-null mutant. These data supported the idea that UCP possesses E3 ubiquitin ligase activity and forms polyubiquitin chains on specific lysines of its substrates.

We repeatedly observed that UCP polyubiquitinates itself and pVHL, and these observations led us to address how the UBC domain exerts catalytic activity to form polyubiquitin chains. The UBC domain harbors two cysteine residues, namely, Cys95 and Cys118. In the case of many subsets of E2 proteins, Cys95 plays a role in ubiquitin charging via a thioester bond [18], and Cys118 has been reported as a structurally important residue related to the donor ubiquitin-binding process in UCP [29]. However, we hypothesized that Cys118 plays a role not only in ubiquitin carrying but also in polyubiquitin elongation because of the E3 ligase activity of UCP. To investigate this hypothesis, we generated a UCP^C118A^ mutant and then compared its catalytic activity to that of wild-type UCP. Interestingly, our results showed that the UCP^C118A^ mutant could not form polyubiquitin chains but accumulated mono- and di-ubiquitin chains, even after a long reaction time (Fig 5A and 5B). Because Fig 1 suggested that UCP ubiquitinated itself in the *trans* manner, we speculated that the catalytic part of UCP transferred ubiquitin to the substrate partly via coordination of Cys95 and Cys118 (Fig 5D, upper panel). We suggest a new mechanism for polyubiquitin chain reaction by UCP, in which an ubiquitin molecule is transferred from Cys95 to Cys118 by trans-thiolation and polyubiquitin chains are formed by reversible thioester bond formation, after which the polyubiquitin chains are moved to the lysine residues of substrate by irreversible isopeptide bonding. Isopeptide bonding was observed on lysine residues and was stable under reducing conditions, whereas the thioester bonding at Cys118 was unstable under these conditions (Fig 5D, lower panel). These results indirectly explain how UCP can play a role as an E3 ubiquitin ligase without a HECT or RING domain. Because thioester bonds are reversible linkages, an ubiquitin on Cys95 can probably be passed to Cys118 via a trans-thiolation reaction. Levin and co-workers demonstrated that the cysteines of E2 proteins can link to a polyubiquitin chain by thioester bonds and can transfer the chain to a cysteine of an E3 ligase via a trans-thiolation process. This report suggested a mechanism in which the growing chain is reciprocally transferred between the active sites of E2 and E3 and polyubiquitin chain synthesis can occur before lysine substrate modification [17]. Moreover, we observed that two or more active Cys118 residues are required when UCP forms polyubiquitin chains (Fig 6). The data showed that UCP interacted with itself and catalyzed a reaction with a substrate leading us to perform an *in vitro* ubiquitination assay using a different form of UCP. When all Cys118 residues of the interacting UCPs were active, polyubiquitin chains formed on the lysine residues of the substrate, whether UCP itself or pVHL (S9 Fig) However, in an interacting form of UCP with one inactive Cys118, polyubiquitin chains did not form; rather, mono- and di-ubiquitin chains accumulated. These data suggested that the elongation of the ubiquitin chain required an active Cys118 residue in both parts of UCP, namely, the catalytic enzyme and the substrate. Collectively, these results suggested that UCP formed polyubiquitin chain on the lysine residues of its substrates in a *trans* manners via the transfer ubiquitin by co-ordination of Cys95 and Cys118 *in-cis* and the -*trans* manners. Furthermore, Cys118 was critical in the dynamic elongation of ubiquitin.

## Conclusion

We found that a member of the ubiquitin-conjugating enzyme family, E2-EPF UCP, formed polyubiquitin chains on lysine residues via ubiquitin transfer through the coordination of Cys95 and Cys118 in *cis* and *trans* manners. Furthermore, Cys118 was critical in the dynamic elongation of ubiquitin on substrate lysine residues in a *trans* manner.

## Supporting Information

S1 Fig Polyubiquitin chains are detected differentially depending on the type of antibody. The lysine-specific linkage of UCP was defined using lysine-to-arginine ubiquitin mutants (K6R, K11R, K48R and K63R), single-lysine ubiquitin mutants (K6, K11, K48 and K63) and lysine-null ubiquitin mutant (K-null). Autoubiquitination assays were performed using GST-UCP (0.2 μg) and ubiquitin or ubiquitin mutants (1.25 μg) at 37°C for 1 h in reaction buffer. The ubiquitinated proteins were detected by immunoblotting using two different ubiquitin antibodies (Santa Cruz, #sc-8017 and Cell Signaling, #3933).
(TIF)

S2 Fig The N-terminal domain of UCP contains lysine residues for polyubiquitination *in vivo*. Each truncated UCP mutant (5 μg) and HA-Ubiquitin (2 μg) were co-transfected into HEK-293T cells. The cells were treated with/without 10 μM MG132 for 12 h, harvested at 48 h post-transfection and pulled down with GST agarose. The ubiquitinated domains were detected by immunoblotting.
(TIF)

S3 Fig Stable knockdown cell lines generated using the UCP shRNA plasmid to deplete endogenous UCP. HeLa cells were co-transfected with shRNA-control (5 μg) or shRNA-UCP (5 μg) and pTK (5 μg) plasmid. After a 24-h incubation, the cells were selected with 500 μg/ml hygromycin B for 2 weeks. The cells were isolated, and hygromycin-selected cells were analyzed by SDS-PAGE using a UCP-specific antibody.
(TIF)

S4 Fig UCP produces polyubiquitin chains on pVHL under denaturing and non-denaturing conditions. *In vitro* pVHL ubiquitination was performed using E1 (0.5 μg), Flag-ubiquitin (1.25 μg), GST-UCP (0.2 μg) and His-VHL protein (2 μg) at 37°C for 1 h in reaction buffer. To isolate ubiquitinated VHL, total reaction mixtures were incubated with Ni-NTA agarose on a rotary shaker at 4°C for 2 h. The beads were then washed three times under reducing (4M Urea, 1% NP40 in NET gel buffer) or non-reducing conditions (1% NP40 in NET gel buffer) and then resuspended in 2X SDS sample buffer under denaturing conditions (β-mercaptoethanol). Polyubiquitin chains on pVHL were detected by immunoblotting using anti-Flag antibody.
(TIF)

S5 Fig Schematic illustration of lysine residues in UCP and pVHL. (A) The UBC domain of UCP is rich in lysine residues; therefore, UCP mutants were generated containing lysine-to-arginine substitutions in the UBC domain. (B) pVHL single-lysine mutants and lysine-null mutant were also investigated.
(TIF)

S6 Fig UCP forms strong isopeptide bond at Lys76 with itself as the substrate. UCP lysine-to-arginine mutants (K18R, K32R, K63R, K68R, K76R, K82R, K100R or K117R) were constructed. *In vitro* autoubiquitination assays were performed using His-UCP^WT^ (0.2 μg) and the UCP lysine mutants (0.2 μg) at 37°C for 1 h. The ubiquitinated forms were analyzed by immunoblotting using anti-Flag antibody.
(TIF)

S7 Fig Schematic structure of UCP. The locations of Lys76, Lys100, Cys95 and Cys118 were indicated on the 3D structure of E2-EPF UCP, supplied by the NCBI protein structure DB (PDB-1ZDN).
(TIF)

S8 Fig An active Cys118 in both interacting partners is essential for polyubiquitin chain formation. (A) *In vitro* ubiquitination assays were performed using GST-UCP^∆N^ (each 0.2 μg, 0.5 μg) and His-UCP^C95A^ or His-UCP^CA^ (2 μg). After the reaction, His-UCP^C95A^ or His-UCP^CA^ was pulled down with Ni-NTA agarose, and polyubiquitination was analyzed by immunoblotting using anti-Flag antibody. (B) Illustration of the expected reaction steps during polyubiquitin chain formation by two different UCP complexes: UCP^∆N^/UCP^C95A^ and UCP^∆N^/UCP^CA^. When a polyubiquitin chain is tethered onto Cys118 (asterisk) by thioesterification, the intermolecular association of Cys118 residues is required to assemble high-molecular-weight ubiquitin chains. The assembled polyubiquitin chain is then linked to lysine residues on the substrate.
(TIF)

S9 Fig Schematic illustration of pVHL ubiquitination. Illustration of the expected reaction steps during pVHL polyubiquitin by two different UCP complexes: UCP^∆N^/UCP^C95A^. Autoubiquitination is occurred by the intermolecular association of Cys118 and the assembled polyubiquitin chain is transferred to lysine residues on the pVHL in a *trans* manner.
(TIF)
