# Supplementary figures and images for: E2-EPF UCP Possesses E3 Ubiquitin Ligase Activity via Its Cysteine 118 Residue
Source: PLoS One. 2016 Sep 29;11(9):e0163710. doi: 10.1371/journal.pone.0163710 (PMC5042379; doi:10.1371/journal.pone.0163710)

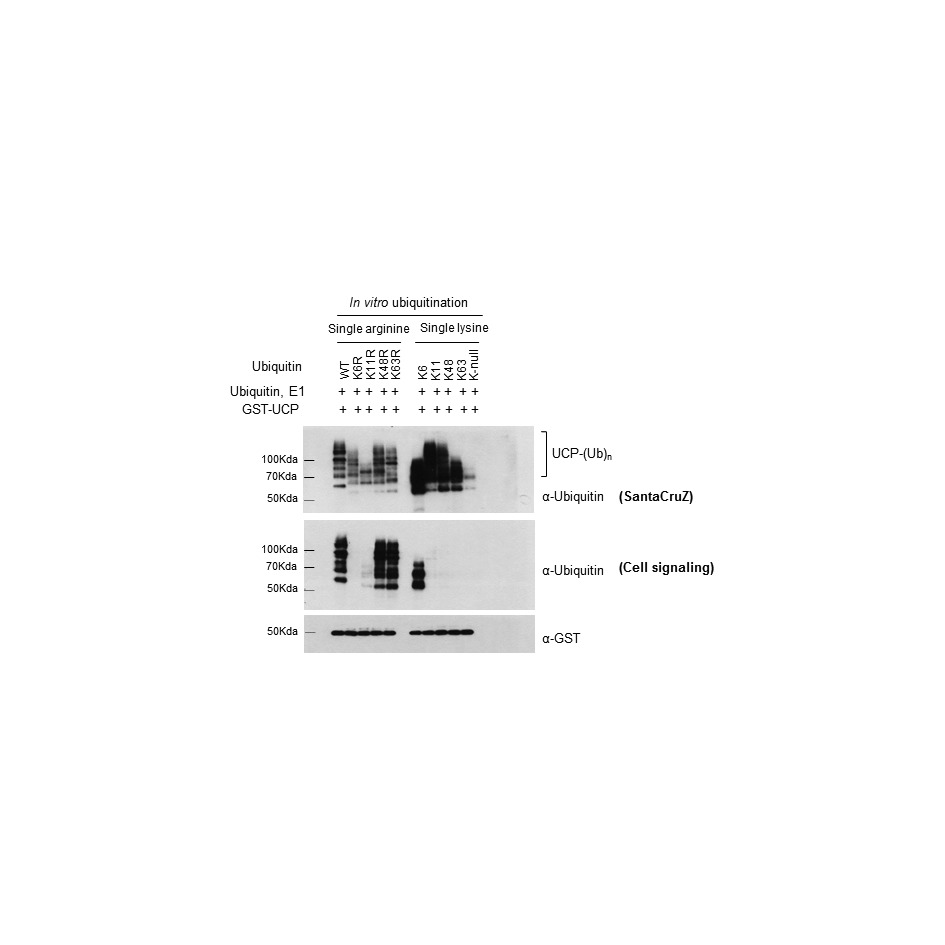

Supplement: S1 Fig — The lysine-specific linkage of UCP was defined using lysine-to-arginine ubiquitin mutants (K6R, K11R, K48R and K63R), single-lysine ubiquitin mutants (K6, K11, K48 and K63) and lysine-null ubiquitin mutant (K-null). Autoubiquitination assays were performed using GST-UCP (0.2 μg) and ubiquitin or ubiquitin mutants (1.25 μg) at 37°C for 1 h in reaction buffer. The ubiquitinated proteins were detected by immunoblotting using two different ubiquitin antibodies (Santa Cruz, #sc-8017 and Cell Signaling, #3933). (TIF) [file pone.0163710.s001.tif]

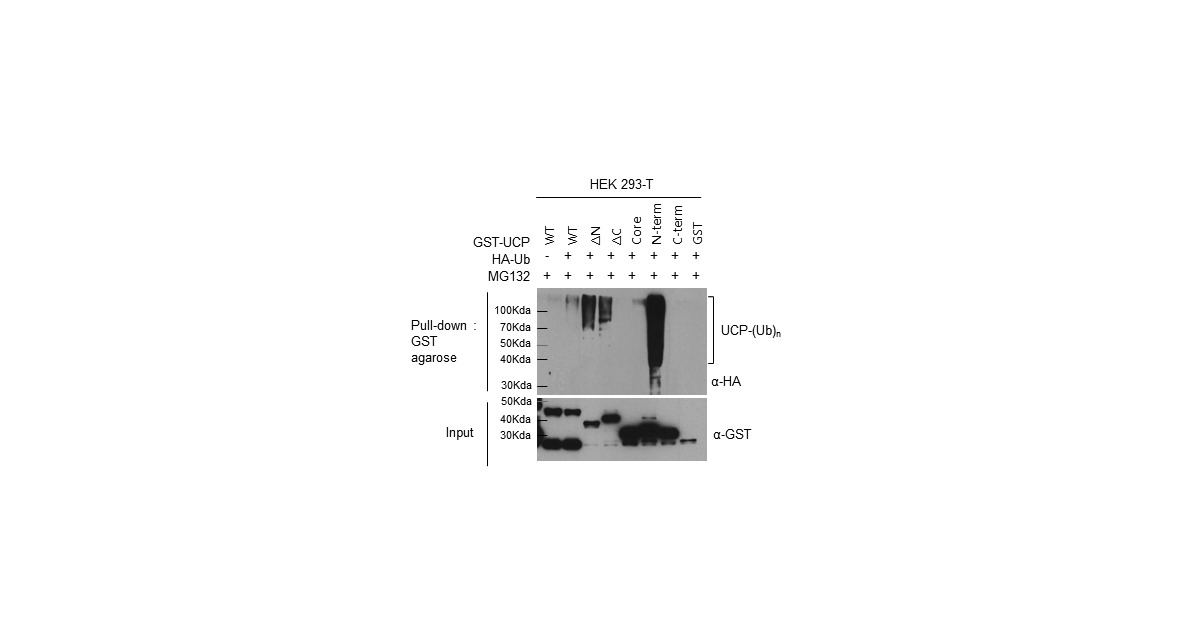

Supplement: S2 Fig — Each truncated UCP mutant (5 μg) and HA-Ubiquitin (2 μg) were co-transfected into HEK-293T cells. The cells were treated with/without 10 μM MG132 for 12 h, harvested at 48 h post-transfection and pulled down with GST agarose. The ubiquitinated domains were detected by immunoblotting. (TIF) [file pone.0163710.s002.tif]

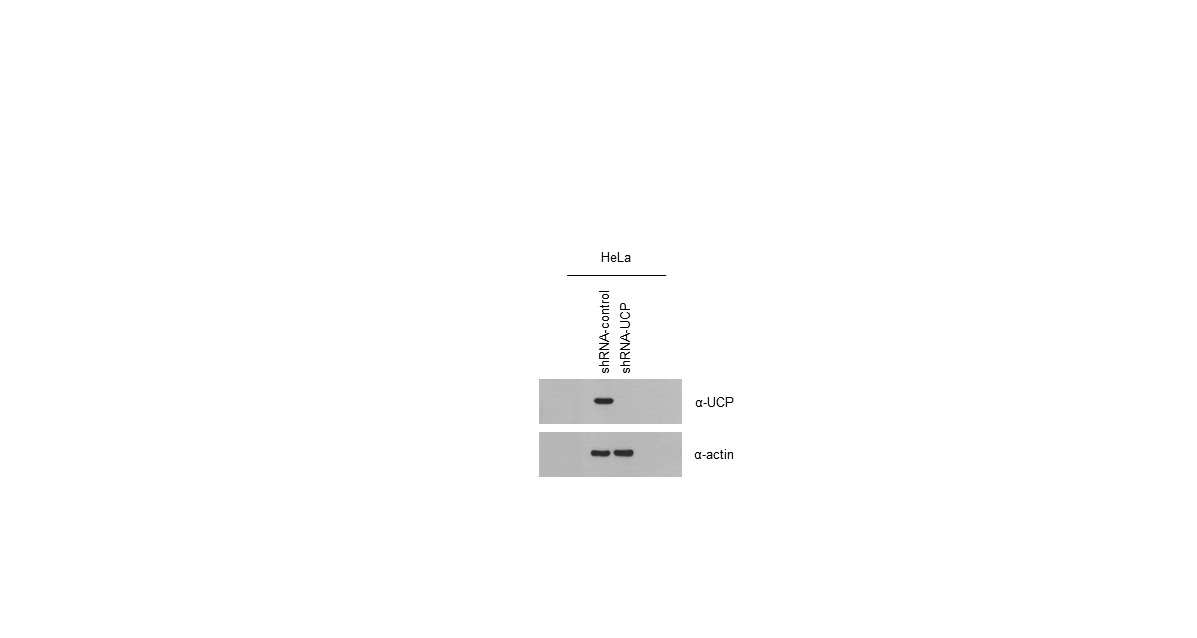

Supplement: S3 Fig — HeLa cells were co-transfected with shRNA-control (5 μg) or shRNA-UCP (5 μg) and pTK (5 μg) plasmid. After a 24-h incubation, the cells were selected with 500 μg/ml hygromycin B for 2 weeks. The cells were isolated, and hygromycin-selected cells were analyzed by SDS-PAGE using a UCP-specific antibody. (TIF) [file pone.0163710.s003.tif]

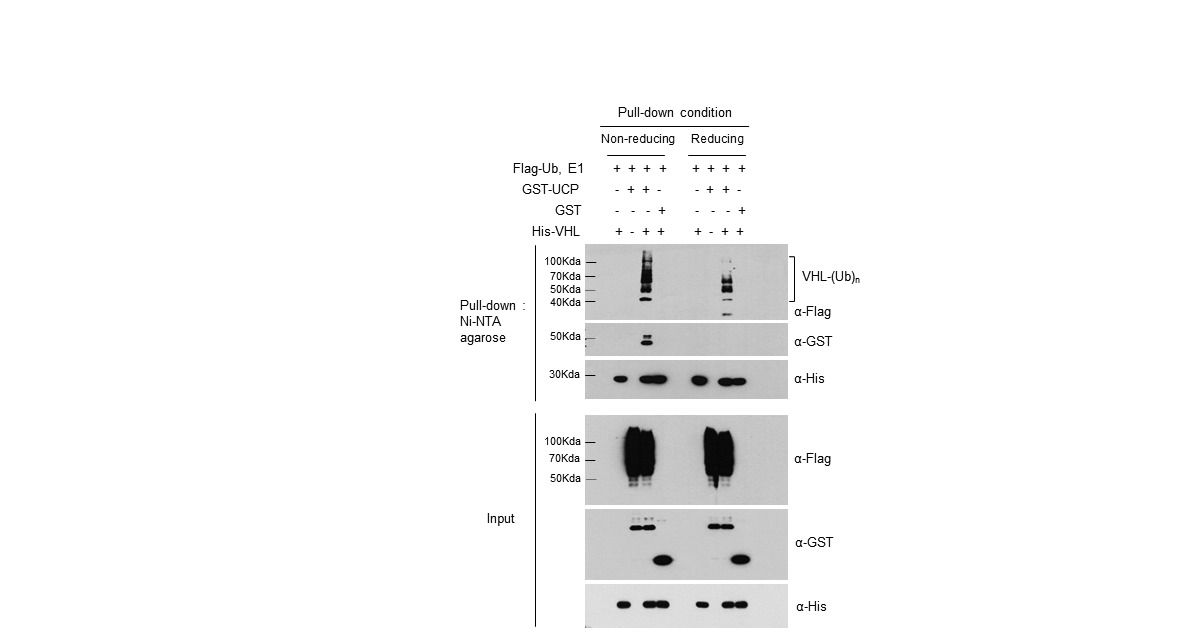

Supplement: S4 Fig — In vitro pVHL ubiquitination was performed using E1 (0.5 μg), Flag-ubiquitin (1.25 μg), GST-UCP (0.2 μg) and His-VHL protein (2 μg) at 37°C for 1 h in reaction buffer. To isolate ubiquitinated VHL, total reaction mixtures were incubated with Ni-NTA agarose on a rotary shaker at 4°C for 2 h. The beads were then washed three times under reducing (4M Urea, 1% NP40 in NET gel buffer) or non-reducing conditions (1% NP40 in NET gel buffer) and then resuspended in 2X SDS sample buffer under denaturing conditions (β-mercaptoethanol). Polyubiquitin chains on pVHL were detected by immunoblotting using anti-Flag antibody. (TIF) [file pone.0163710.s004.tif]

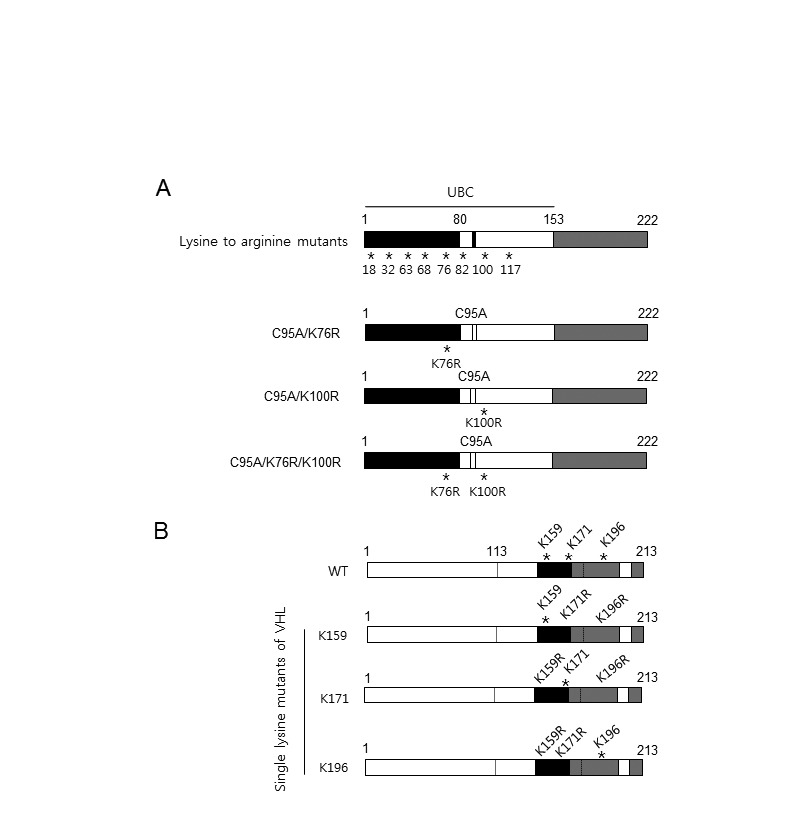

Supplement: S5 Fig — (A) The UBC domain of UCP is rich in lysine residues; therefore, UCP mutants were generated containing lysine-to-arginine substitutions in the UBC domain. (B) pVHL single-lysine mutants and lysine-null mutant were also investigated. (TIF) [file pone.0163710.s005.tif]

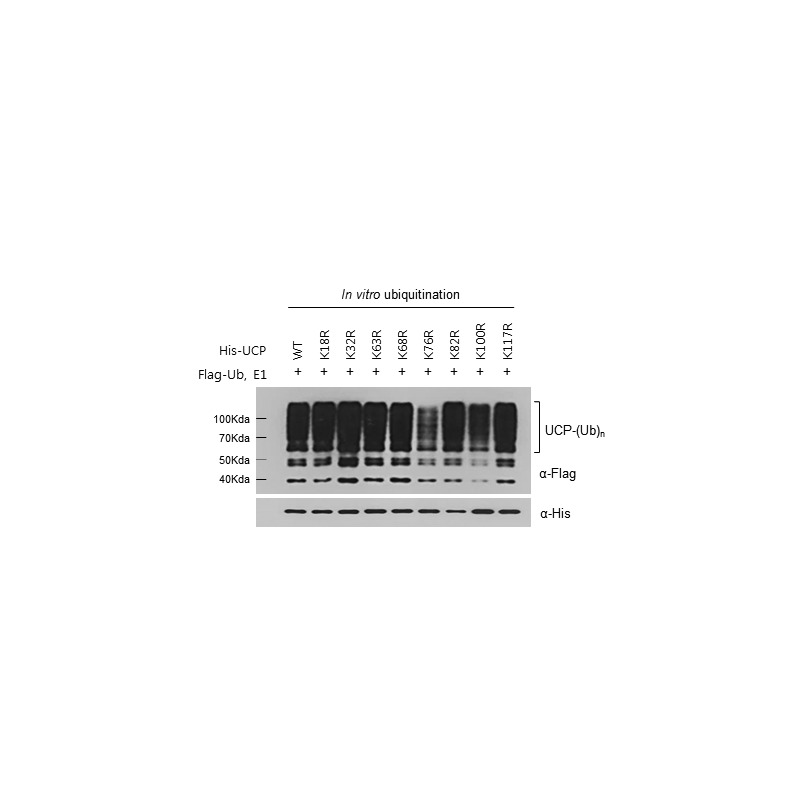

Supplement: S6 Fig — UCP lysine-to-arginine mutants (K18R, K32R, K63R, K68R, K76R, K82R, K100R or K117R) were constructed. In vitro autoubiquitination assays were performed using His-UCPWT (0.2 μg) and the UCP lysine mutants (0.2 μg) at 37°C for 1 h. The ubiquitinated forms were analyzed by immunoblotting using anti-Flag antibody. (TIF) [file pone.0163710.s006.tif]

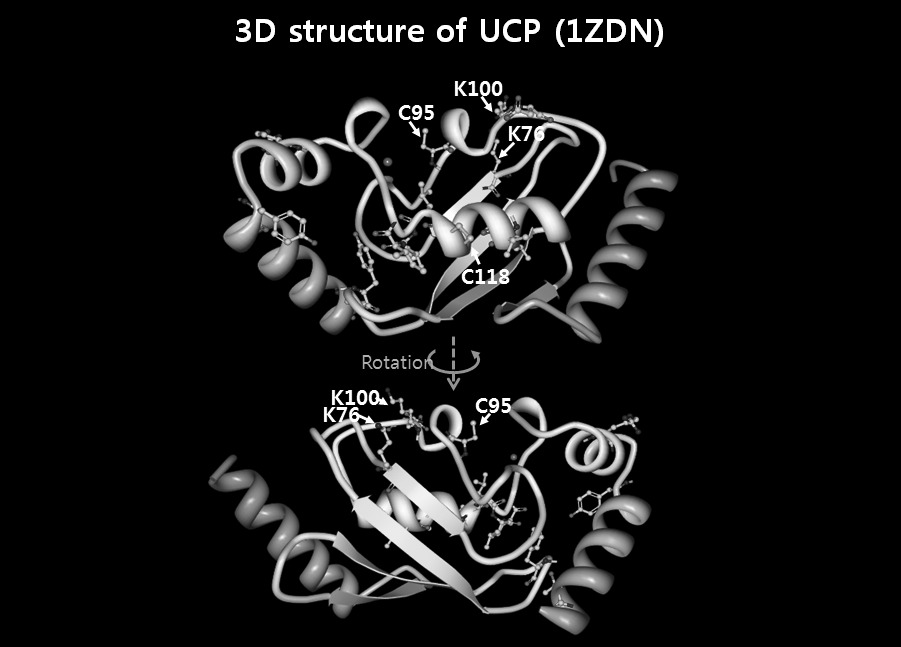

Supplement: S7 Fig — The locations of Lys76, Lys100, Cys95 and Cys118 were indicated on the 3D structure of E2-EPF UCP, supplied by the NCBI protein structure DB (PDB-1ZDN). (TIF) [file pone.0163710.s007.tif]

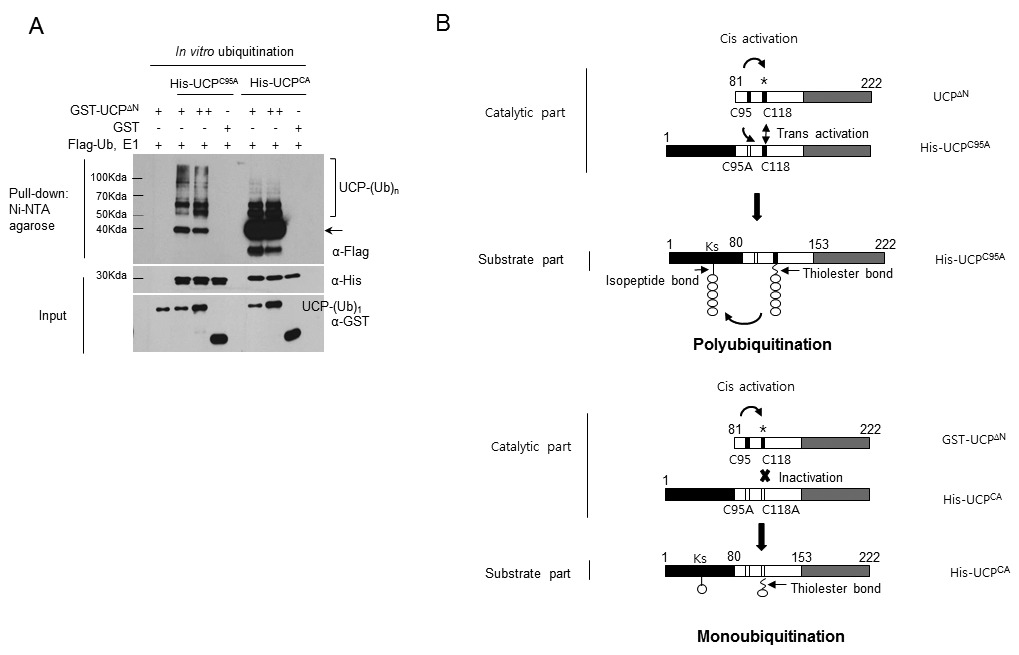

Supplement: S8 Fig — (A) In vitro ubiquitination assays were performed using GST-UCP∆N (each 0.2 μg, 0.5 μg) and His-UCPC95A or His-UCPCA (2 μg). After the reaction, His-UCPC95A or His-UCPCA was pulled down with Ni-NTA agarose, and polyubiquitination was analyzed by immunoblotting using anti-Flag antibody. (B) Illustration of the expected reaction steps during polyubiquitin chain formation by two different UCP complexes: UCP∆N/UCPC95A and UCP∆N/UCPCA. When a polyubiquitin chain is tethered onto Cys118 (asterisk) by thioesterification, the intermolecular association of Cys118 residues is required to assemble high-molecular-weight ubiquitin chains. The assembled polyubiquitin chain is then linked to lysine residues on the substrate. (TIF) [file pone.0163710.s008.tif]

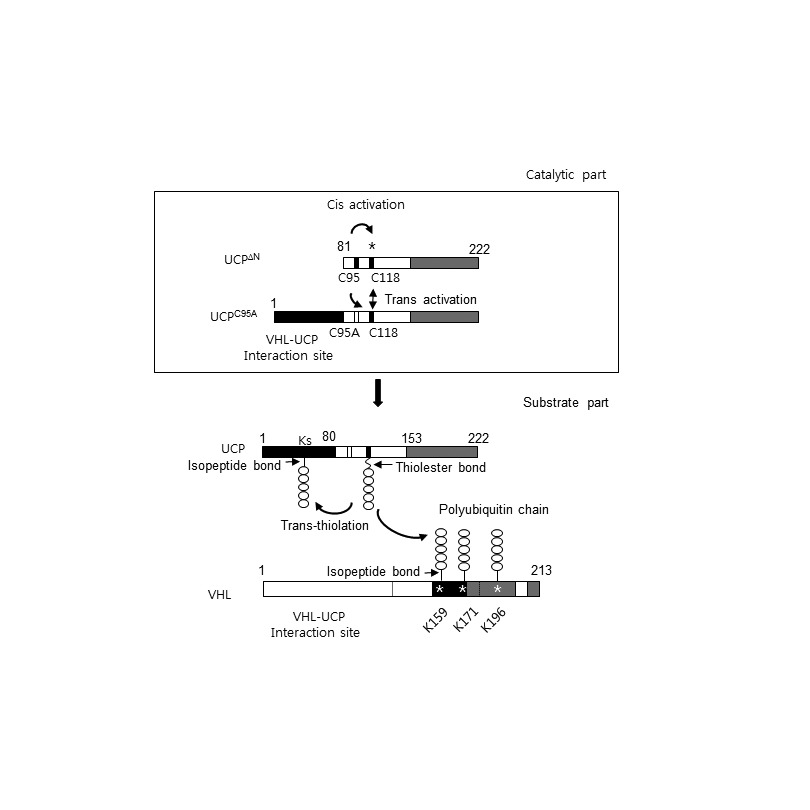

Supplement: S9 Fig — Illustration of the expected reaction steps during pVHL polyubiquitin by two different UCP complexes: UCP∆N/UCPC95A. Autoubiquitination is occurred by the intermolecular association of Cys118 and the assembled polyubiquitin chain is transferred to lysine residues on the pVHL in a trans manner. (TIF) [file pone.0163710.s009.tif]
